# Will (or can) people pay for headache care in a poor country?

**DOI:** 10.1007/s10194-011-0398-1

**Published:** 2011-11-02

**Authors:** Christian Lampl, Timothy Joseph Steiner, Thomas Mueller, Eka Mirvelashvili, Mamuka Djibuti, Maka Kukava, Anna Dzagnidze, Rigmor Jensen, Lars Jacob Stovner, Zaza Katsarava

**Affiliations:** 1 Department of Neurology, University of Essen, Hufelandstrasse 55, 45122 Essen, Germany; 2 Department of Neurology and Pain Medicine, Upper Austrian Headache Centre, Konventhospital Barmherzige Brüder, Linz, Austria; 3 Department of Neuroscience, Norwegian National Headache Centre, Norwegian University of Science and Technology, St. Olav’s University Hospital, Trondheim, Norway; 4 Department of Neuroscience, Imperial College London, London, UK; 5 Department of Laboratory Medicine, Konventhospital Barmherzige Brüder, Linz, Austria; 6 School of Public Health, Tbilisi State Medical University, Tbilisi, Georgia; 7 NGO: “Partnership in Research and Action for Health”, Tbilisi, Georgia; 8 Department of Neurology, Tbilisi Medical University, Tbilisi, Georgia; 9 Department of Neurology, Danish Headache Centre, Glostrup Hospital, University of Copenhagen, Copenhagen, Denmark; 10 St. Olav’s University Hospital, Trondheim, Norway

**Keywords:** Headache-related health care, Burden of disease, Quality of life, Health economics, Willingness to pay, Republic of Georgia

## Abstract

We asked whether attempts to introduce headache services in poor countries would be futile on grounds of cost and unsustainability. Using data from a population-based survey in the Republic of Georgia, an exemplary poor country with limited health care, and against the background of headache-attributed burden, we report on willingness to pay (WTP) for effective headache treatment. Consecutive households were visited in areas of Tbilisi (urban) and Kakheti (rural), together representative of Georgian habitation. Biologically unrelated adults were interviewed by medical residents using a structured ICHD-II-based diagnostic questionnaire, the MIDAS questionnaire and SF-36. The bidding-game method was employed to assess WTP. Of 1,145 respondents, 50.0% had episodic headache (migraine and/or tension-type headache) and 7.6% had headache on ≥15 days/month, which was not further diagnosed. MIDAS scores were higher in people with headache on ≥15 days/month (mean 11.2) than in those with episodic headache (mean 7.0; *P* = 0.004). People with headache had worse scores in all SF-36 sub-scales than those without, but no differences were found between headache types. Almost all (93%) respondents with headache reported WTP averaging USD 8 per month for effective headache treatment. WTP did not correlate with headache type or frequency, or with MIDAS or SF-36 scores. Headache is common and headache-attributed burden is high in Georgia, with a profound impact on HRQoL. Even those less affected indicated WTP for effective treatment, if it were available, that would on average cover costs, which locally are low. Headache services in a poor country are potentially sustainable.

## Introduction

Primary headache disorders, in particular migraine and tension-type headache (TTH), are common [1]. They cause substantial disability amongst those affected and impose secondary burdens on their families and work colleagues [2]. Through lost productivity, they generate a very large economic burden that falls upon society [3]. Throughout the world, medical care for people with headache generally fails to alleviate these burdens. In most developing countries, for example many in Eastern Europe including the Republic of Georgia, primary headaches are altogether neglected by health policy-makers, physicians and even by some people affected by them, and treatment of headache is not at all a part of medical care.

The ultimate objective of the Global Campaign against Headache [4, 5] is to support the implementation of effective headache services to meet locally assessed needs, thereby reducing the burden of headache. This is challenging in a resource-limited world. Headache services are manifestly not cost-free, and other priorities compete. In wealthy economies, the high financial cost of headache disorders argues strongly for greater investment in headache services since lost-productivity costs [3] are far higher than service costs [6]. Is this also the case in poor countries, or are attempts to introduce headache services in poor countries merely an exercise in optimistic and well-meaning futility on grounds of unsustainability?

The East European Republic of Georgia is an exemplary (for our purposes) poor country. In the post-Soviet era, social infrastructure is unsound, incomes for the majority of people are low and health services, generally patchy, are effectively non-existent for headache. A recent population-based survey of the prevalence and burden of primary headaches in Georgia [7] found levels of migraine (MIG) and tension-type headache (TTH) in line with estimates from other parts of the world [1]. What was unusual was a very high prevalence (7.6%) of headache occurring on ≥15 days/month, which was strongly associated with low socio-economic status. Clearly, headache is very common in Georgia and the needs of people affected by it are not adequately met.

We had the opportunity, during this survey, to seek an answer to the question we pose above. Using data from the survey, we report on headache-attributed burden and health-related quality of life (HRQoL) among people with headache in Georgia as indices of need. In addition, and as our principal purpose, we report on people’s willingness to pay (WTP) for effective headache treatment, if it were available in the country.

## Methods

The Georgian National Council on Bioethics approved the study protocol. All respondents were informed of the purpose of the survey and gave their verbal consent prior to participating.

The methods of the study, conducted during 2008, have been reported in full previously [8]. Briefly, four medical residents, trained in understanding and applying the diagnostic criteria for primary headache disorders, visited 500 adjacent households in Tbilisi and 300 in Kakheti, the areas selected being, respectively, representative of urban and rural Georgian habitation. They interviewed all 1,701 adults living in these households, selecting, for the study, husband and wife and any other biologically unrelated adults (*n* = 1,145). A screening question asked whether headache, “not related to flu, hangover, cold or head injury”, had occurred at least once within the previous year. When it had, further questions separated episodic headache from headache occurring on ≥15 days/month. A previously validated structured diagnostic questionnaire [8] based on the International Classification of Headache Disorders, 2nd edition (ICHD-II) [9] was used to diagnose MIG and/or TTH in cases of episodic headache. Cases of headache occurring on ≥15 days/month were not further diagnosed for this analysis but kept separate as a single group.

Headache-attributed burden was assessed using the Migraine Disability Assessment (MIDAS) questionnaire [10], recording the numbers of lost days of school or paid work, household work and family, social or leisure activities during the previous 3 months because of headache. MIDAS score is derived as the sum of wholly lost days and days of reduced productivity by >50%; any resulting over-estimate is balanced by the under-estimate that arises from ignoring days impaired by <50% [10].

HRQoL was assessed using the 36 Short Form (SF-36) health survey questionnaire [11], a widely used generic instrument developed by the Rand Corporation for the Medical Outcomes Study, which has been tested, validated and used in many chronic diseases including headache [12]. SF-36 is a self-administered 36-item scale measuring eight domains of health including physical functioning (PF), role limitations due to physical problems (RP), bodily pain (BP), general health (GH), vitality (VT), social functioning (SF), role limitations due to emotional problems (RE) and mental health (MH) during the preceding 4 weeks. All items are scored 0 to 100, with a higher score indicating better health. The eight domains are aggregated into two higher order scores that measure the physical component of HRQoL (Physical Component Summary, or PCS) and the mental component (Mental Component Summary, or MCS).

WTP was assessed by the bidding-game method [13]. Interviewers asked respondents with headache how much money (in Georgian lari [GEL]) they would spend per month for an effective medication package, which was defined as treatment achieving “very good” pain relief for acute headache coupled with preventative medication reducing headache frequency by more than one half. The bidding began by first asking whether the individual would pay GEL 15 (USD 7.50) for the package. If the answer was “yes”, the interviewer incremented the bid in steps of GEL 5 (USD 2.50) until the answer was “no”, and the last sum receiving a “yes” response was the WTP. If the initial answer was “no”, the interviewer reduced the bid by GEL 5 (USD 2.50) until the respondent said “yes”, the first sum receiving this response then being the WTP.

Respondents’ wealth was assessed by interviewers as low, intermediate or high based on impressions of the area and quality of housing, possessions and apparent style of living. For this analysis, those of low wealth (“poorer”) were compared with those of intermediate and high wealth combined (“wealthier”), as few were in the high-wealth group.

## Analysis

The outcome variables of the study were MIDAS scores, scores for SF-36 sub-scales and PCS and MCS, and WTP expressed in US dollars (USD). Comparisons were made between episodic headache and headache occurring on ≥15 days/month, and between MIG (including definite [dMIG] and probable [pMIG]) and TTH (including definite [dTTH] and probable [pTTH]). For SF-36, comparisons were also possible between headache cases and those with no headache.

Data were statistically analysed with the SPSS 13.0 software (SPSS Inc., Chicago, IL, USA) and the MedCalc 11.3.1.0 package (MedCalc Software, Mariakerke, Belgium). Categorical data were expressed as absolute numbers (percent) and ordinal and metric variables as mean [±standard deviation (SD)] and as median [interquartile range (IQR)]. Comparisons between groups were calculated using the Chi-squared test for categorical data and the Mann–Whitney *U* test or the Kruskal–Wallis test for ordinal and metric variables as appropriate. If the Kruskal–Wallis test was positive (*P* < 0.05), then post hoc analysis for pair-wise comparison of subgroups [14] was performed. All probabilities were two-tailed, and *P* < 0.05 was regarded as statistically significant; *P* values were not adjusted for multiple comparisons and were considered descriptive only.

## Results

Prevalence data have been reported previously [7], and are briefly summarized here. Household response rates were high: 92% (462 of 500) in Tbilisis and 100% (of 300) in Kakheti. In the population-based sample of 1,145 respondents [690 (60%) women, mean age 45.4±12.0 years], 659 (57.6%) had headache not related to flu, hangover, cold or head injury. Of these, 87 (7.6%) had headache on ≥15 days/month. We treated these as a single separate group. Of the 572 (50%) with episodic headache, 157 (13.7%) had MIG and 383 (33.4%) had TTH (these numbers each including 70 respondents who had both). Mainly because of inconsistent responses, 102 (8.9%) cases were unclassifiable. We excluded these from this analysis, and report below on 557 respondents, 470 with episodic headache and 87 with headache on ≥15 days/month.

MIDAS scores were available from 393 people [70.5%; 339 with episodic headache (69 MIG, 236 TTH, 34 MIG + TTH) and 54 with headache on ≥15 days/month] (Table 1). They were higher in those with headache on ≥15 days/month [mean 11.19 (±11.02); median 10] than in those with episodic headache [mean 6.95 (±7.32); median 5; *P* = 0.004] and in those with MIG [mean 9.61 (±8.37); median 10] than in those with TTH [mean 6.03 (±6.85); median 5; *P* = 0.001]. MIDAS grades III and IV (i.e, MIDAS score ≥11), indicating moderate or severe impact, were more common in people with headache on ≥15 days/month (38.9%) than in those with episodic headache (17.7%; χ^2^ = 37.504; *P* < 0.001) (Table 1).

**Table 1:** Headache-related disability assessed by MIDAS in people with episodic headache (MIG or TTH) or with headache occurring on ≥15 days/month

	All episodic headache (*n* = 339)	MIG (*n* = 69)	TTH (*n* = 236)	MIG + TTH (*n* = 34)	Headache on ≥15 days/month (*n* = 54)
Days of missed work or school	1.14 (±1.46)	1.61 (±1.78)	1.03 (±1.37)	0.91 (±1.19)	1.91 (±3.28)
	1 (0–2)	1 (0–2)	1 (0–2)	0 (0–2)	1 (0–2)
Days of work or school with <50% productivity	1.54 (±1.76)	2.07 (±1.78)	1.31 (±1.56)	2.06 (±2.62)	2.50 (±2.52)
	1 (0–2)	2 (1–3)	1 (0–2)	2 (0–2)	2 (0–3)
Days of no household work	1.35 (±1.50)	1.93 (±1.74)	1.19 (±1.40)	1.29 (±1.34)	2.06 (±2.36)
	1 (0–2)	2 (1–3)	1 (0–2)	2 (0–2)	2 (0–3)
Days of household work with <50% productivity	1.56 (±1.77)	2.07 (±1.78)	1.32 (±1.56)	2.24 (±2.63)	2.85 (±3.14)
	1 (0–2)	2 (1–3)	1 (0–2)	2 (0–2)	2 (1–4)
Days of no social/family/leisure activity	1.35 (±1.50)	1.93 (±1.74)	1.17 (±1.38)	1.44 (±1.46)	1.87 (±2.10)
	1 (0–2)	2 (1–3)	1 (0–2)	2 (0–2)	2 (0–3)
Total MIDAS score	6.95 (±7.32)	9.61 (±8.37)	6.03 (±6.85)	7.94 (±6.95)	11.19 (±11.02)
	5 (0–10)	10 (5–15)	5 (0–10)	8 (0–10)	10 (4–15)
MIDAS grade I (%)	52.5	37.7	58.9	38.2	37.0
MIDAS grade II (%)	29.8	33.3	26.3	47.1	24.1
MIDAS grade III (%)	14.5	23.2	12.3	11.8	24.1
MIDAS grade IV (%)	3.2	5.8	2.5	2.9	14.8

Data are shown as mean (±SD) and median (IQR), or as prevalence (%)

*IQR* inter-quartile range, *MIG* migraine, *TTH* tension-type headache

**Fig. 1:**
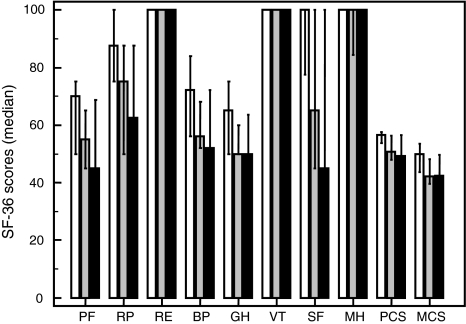
SF-36 domain-specific quality-of-life scores among people with no headache* open square*, episodic headache* gray filled square* or headache on ≥15 days/month* black filled square*. *PF* physical functioning, *RP* role limitations due to physical problems, *RE* role limitations due to emotional problems, *BP* bodily pain, *GH* general health, *VT* vitality, *SF* social functioning, *MH* mental health, *PCS* physical component summary, *MCS* mental component summary

SF-36 data were available from 1,066 respondents with or without headache (93.1%) (Table 2). Figure 1 shows SF-36 domain-specific quality-of-life scores among people with no headache, episodic headache, and headache occurring on ≥15 days/month. People without headache had higher scores in all sub-scales than those with headache, but no differences were found between respondents with episodic headache and those with headache on ≥15 days/month or between those with MIG and those with TTH. No significant correlations were observed between headache frequency (interval-scaled) and any SF-36 sub-scale (data not shown).

**Table 2:** Health-related quality of life assessed by SF-36 in people with no headache, episodic headache or headache occurring on ≥15 days/month

SF-36 domains	No. headache (*n* = 418)	Episodic headache (*n* = 561)	Headache on ≥15 days/month (*n* = 87)	*P* (Kruskal Wallis)
PF	65.8 (±15.8)	55.3 (±15.1)	54.3 (±20.5)	<0.0001^2^
	70 (50–75)	55 (45–65)	45 (40–70)	
RP	83.5 (±18.9)	69.7 (±18.4)	69.0 (±21.0)	<0.0001^2^
	88 (75–100)	75 (50–88)	63 (50–88)	
RE	99.2 (±4.4)	97.2 (±8.8)	98.9 (±2.8)	<0.0001^2^
	100 (100–100)	100 (100–100)	100 (100–100)	
BP	69.5 (±14.8)	59.6 (±13.3)	59.5 (±15.0)	<0.0001^1^
	72 (56–84)	56 (52–68)	52 (52–72)	
GH	65.2 (±14.5)	55.1 (±13.0)	55.5 (±14.4)	<0.0001^1^
	65 (50–75)	50 (50–60)	50 (50–65)	
VT	96.5 (±17.9)	81.9 (±36.0)	86.2 (±33.8)	<0.0001^2^
	100 (100–100)	100 (100–100)	100 (100–100)	
SF	88.9 (±19.5)	69.4 (±24.2)	63.6 (±25.0)	<0.0001^1^
	100 (78–100)	65 (45–100)	45 (45–100)	
MH	96.1 (±18.0)	81.2 (±36.0)	86.1 (±34.1)	<0.0001^2^
	100 (100–100)	100 (84–100)	100 (100–100)	
PCS	55.2 (±4.3)	51.1 (±5.5)	50.7 (±5.5)	<0.0001^1^
	56 (54–58)	51 (48–56)	49 (48–57)	
MCS	48.4 (±7.1)	42.6 (±7.8)	43.0 (±8.2)	<0.0001^1^
	50 (44–54)	42 (40–48)	42 (39–50)	

Post hoc analysis with pairwise comparison of sub-groups according to Conover [14]. Data are shown as mean (±SD) and median (IQR)

*IQR* inter-quartile range, *MIG* migraine, *TTH* tension-type headache

^1^No headache differs from both episodic headache and headache occurring on ≥15 days/month, without significant difference between the last two

^2^No headache differs from episodic headache, without significant difference between episodic headache and headache occurring on ≥15 days/month

Data for WTP were available from 510 (91.6%) people with headache, of whom 93% reported that they would pay a mean of USD 8.1 (±8.7; median USD 5; IQR 5–5) per month for effective treatment if it were available in the country. People in Tbilisi (*n* = 274), the capital, would pay more (mean USD 9.4 ± 9.6; median USD 5; IQR 5–10) per month than those in rural Kakheti (*n* = 236; mean USD 6.5 ± 7.3; median USD 5; IQR 5–5) and wealthier people (*n* = 296) would pay more (mean USD 9.0 ± 9.3; median USD 5; IQR 5–10) than poorer people (*n* = 214; mean USD 6.8 ± 7.7; median USD 5; IQR 5–5). WTP did not correlate with headache type or frequency, or with MIDAS or SF-36 scores (data not shown).

## Discussion

The data contributing to this analysis came from a population-based survey of >1,000 respondents using sound and validated epidemiological methods [7]. Response rates were generally high: in the derivation of the original sample, household response rate was 95% overall, allowing little if any bias, whilst response rates within the sample for SF-36 (93%) and WTP (92%) were also very good. Only for MIDAS (70.5%) was the rate less satisfactory. It may not be compatible with Georgian culture voluntarily to admit that headache causes lost time, or it may be that those who did not lose time saw the questions as pointless (these possible explanations being counter-balancing in whom they would exclude, again making significant bias unlikely). WTP data were collected contemporaneously with other data allowing diagnosis and describing headache-attributed burden. This made possible comparisons between diagnoses, and correlations between WTP and burden.

We diagnosed episodic headache according to ICHD-II [9] as far as was possible, although 102 cases (17.8% of episodic headache cases) were unclassifiable (and excluded from these analyses). Headache occurring on ≥15 days/month might be any of a group of disorders, including chronic MIG, chronic TTH and medication-overuse headache (MOH), that are often but imprecisely referred to collectively as “chronic daily headache”; we preferred to avoid this term. Headache on ≥15 days/month proved very difficult to diagnose by questionnaire, and might in some cases require multiple diagnoses; therefore we analysed all such cases together, as a separate group.

Headache-attributed burden is multidimensional, and extends beyond the person immediately affected to family, friends and work colleagues, and to society as a whole. It is impossible to estimate it in its entirety. We have considered aspects that appear of particular importance—HRQoL and lost productive time. WTP is also an indicator of burden.

SF-36 demonstrated that HRQoL was reduced by headache but this was not quantitatively dependent upon headache type or frequency. The failure of SF-36 to differentiate between episodic headache and headache on ≥15 days/month is particularly surprising, but here is not the place to speculate in detail on the cause. MIDAS, on the other hand, revealed a greater burden from headache occurring on ≥15 days/month than from episodic headache, which is expected.

HRQoL reflects people’s assessment of their general well-being and position in life as perceived within the context of their culture, value systems, goals and concerns [15]. SF-36 is the most widely used HRQoL questionnaire in patients with chronic diseases [11, 15] and has been utilized in several epidemiological and clinic-based studies on headache, all agreeing that people with MIG have lower SF-36 scores than population controls [2, 17]; in one, people with moderate to high disability from MIG had lower HRQoL scores in all SF-36 domains [2] suggesting a profound impact on HRQoL. Other studies have looked at other headache types in addition to MIG [18–20], always finding HRQoL to be negatively affected.

The MIDAS Questionnaire, on the other hand, reflects disability. Originally designed as a screening instrument for people severely affected by headache, who might most benefit from medical care, and to provide an outcome measure for clinical practice [21–26], clinical trials [24, 27–31] and epidemiologic studies [10, 18, 32–39], it is a measure of behavioural response to impairment rather than of disability itself, producing scores expressing lost useful time. Like HRQoL measures, it is intended to aggregate the impact of illness on an individual over a period of time; unlike SF-36, it is disease specific, although applicable to headache rather than only to MIG [40]. It is essentially sensitive to frequency, which may explain why, in our study, MIDAS detected a relationship between headache frequency and burden while SF36 did not. Being disease specific, MIDAS cannot be applied to non-headache controls, who, of course, lose no time from headache. In other words, attribution to headache is explicit in the case of MIDAS, but implicit and inferred from comparisons with non-headache controls in the case of SF-36.

Together, these measures and the prevalence data reveal a population burdened by headache, with unmet need for health care. This is the context in which we explored WTP.

The results of the WTP enquiry are of interest for several reasons. First, we found that >90% of people with headache were willing to pay, out of their pockets from their generally very limited resources, for effective headache care. This is a striking argument against the view that headache is a problem of wealthy countries and unimportant in low-income countries such as Georgia. Second, while WTP varied with financial means (results not shown) as might be expected, it did so within a relatively narrow range (75% of both wealthier and poorer groups would pay between USD 5 and USD 10). This suggests that WTP is driven strongly by need, and only within limits by ability to pay, which is an important finding. However, our method of assessing wealth was inexact, because income in Georgian families is frequently hidden, or provided by a family member who lives and works outside the country and therefore not registered. Direct questions about income are not welcome, and responses would not necessarily reflect reality. Furthermore, in the rapid transition to a US-style market economy, many people with university education lost their jobs and became poor, while others, mostly young people, have prospered better even without education. Therefore, normal socioeconomic indicators do not work well, and so this was an imprecise analysis. Third, WTP did not depend on headache type or frequency. This demonstrates that people with less-frequent headaches are similarly interested in treating them to those with near-daily headache, and strongly suggests that a headache service, if available in the country, would be used not only by the minority of people with headache on ≥15 days/month but rather by the entire population of headache sufferers, seeking to reduce their personal burden of headache and to have a better quality of life. Fourth, of course all depends on alignment between WTP and the actual cost of the service. Whilst a WTP of USD 8 per month might seem rather low compared with what is often spent in western countries, it would be sufficient for many people when set against the low general costs in Georgia. Fifth, since not only the wealthy but also people with low income were willing to pay at least something, we believe these results are very important for market analysis by the pharmaceutical industry, who should have an interest in introducing modern anti-migraine drugs (e.g., triptans) to the Georgian market. The importance of this is in the following: without effective drugs, headache services will remain limited, and, while headache services are limited, the market for effective drugs will remain depressed. This Catch-22 situation needs urgently to be breached.

There is one caveat: we have recorded what people *say* they would do in the (currently) abstract circumstances of available good care; it is not certain, until empirically tested, which we hope later to do, that they will actually *do* it when the opportunity becomes real. Regardless of this, and a sixth point of interest, WTP is an expression of burden that probably captures more elements of it than either HRQoL or disability measures.

We have something more to say about headache care in Georgia. During the survey, we received the impression that headache was not considered, by those affected, an important medical problem. The majority of respondents considered their headaches, whether MIG or TTH, to be a natural part of their lives; people were surprised to learn that headache could be treated effectively. Many were very enthusiastic to hear that efforts were being made by *Lifting The Burden* [4, 5] to establish a headache service in the country. Funding of medical care in Georgia is a major issue. In the Soviet era, the State guaranteed the necessary minimum of wealth, and basic health-care services were provided with no out-of-pocket payments. Transition from this socialist system to a market economy has been accompanied by a marked socioeconomic decline for many inhabitants, and the development of significant disparities. Most people are not insured, and all costs for any treatment are out-of-pocket expenses.

In these circumstances, would people, in a poor country, pay for a service for primary headache disorders, which are not life-threatening but “only” reduce quality of life? We have found the answer to be “yes”, with the caveat referred to earlier. We assume that the answer would be the same in other similarly poor countries, and believe that efforts to introduce headache services in such countries are far from futile, and must be continued.
